# Analysis of Alcohol Industry Submissions against Marketing Regulation

**DOI:** 10.1371/journal.pone.0170366

**Published:** 2017-01-24

**Authors:** Florentine Petronella Martino, Peter Graeme Miller, Kerri Coomber, Linda Hancock, Kypros Kypri

**Affiliations:** 1 School of Psychology, Faculty of Health, Deakin University, Geelong, Australia; 2 School of Humanities and Social Sciences, Faculty of Arts & Education, Deakin University, Melbourne, Australia; 3 School of Medicine and Public Health, University of Newcastle, Newcastle, Australia; 4 Injury Prevention Research Unit, Department of Preventive and Social Medicine, University of Otago, Dunedin, New Zealand; Pennsylvania State University College of Medicine, UNITED STATES

## Abstract

A growing body of literature points to the role of vested interests as a barrier to the implementation of effective public health policies. Corporate political activity by the alcohol industry is commonly used to influence policy and regulation. It is important for policy makers to be able to critique alcohol industry claims opposed to improved alcohol marketing regulation. The Australian National Preventive Health Agency reviewed alcohol marketing regulations in 2012 and stakeholders were invited to comment on them. In this study we used thematic analysis to examine submissions from the Australian alcohol industry, based on a system previously developed in relation to tobacco industry corporate political activity. The results show that submissions were a direct lobbying tactic, making claims to government that were contrary to the evidence-base. Five main frames were identified, in which the alcohol industry claimed that increased regulation: (1) is unnecessary; (2) is not backed up by sufficient evidence; (3) will lead to unintended negative consequences; and (4) faces legal barriers to implementation; underpinned by the view (5) that the industry consists of socially responsible companies working toward reducing harmful drinking. In contrast with tobacco industry submissions on public policy, which often focused on legal and economic barriers, the Australian alcohol industry placed a heavier emphasis on notions of regulatory redundancy and insufficient evidence. This may reflect differences in where these industries sit on the ‘regulatory pyramid’, alcohol being less regulated than tobacco.

## Introduction

Exposure to marketing of alcoholic beverages is associated with increased alcohol consumption, especially in young people, and contributes to earlier initiation of alcohol use, the development of positive and carefree attitudes toward drinking in the general public, and alcohol-related violence [1, 2]. Similar associations have been found for tobacco; the marketing of which increases the likelihood that adolescents start to smoke [3, 4].

### Current Alcohol Marketing Regulation

In Australia, a quasi-regulatory framework is in place to protect against potential harmful effects of alcohol marketing on children and youth. At the centre of this is the Alcohol Beverages Advertising (and Packaging) Code Scheme (ABAC), which monitors and responds to complaints about the content of alcohol marketing. It consists of three elements: a self-regulatory alcohol marketing code; a pre-vetting service; and a public complaints and adjudication panel. These are overseen by a six-member management committee, comprised of three alcohol industry representatives, an advertising industry representative, a government representative and, as of July 2015, an Independent Chair [5]. Compliance with the scheme is voluntary, i.e., there are no legal or pecuniary sanctions for violation of the code. Other relevant codes that cover some content restrictions include the Australian Association of National Advertisers (AANA) Code of Ethics and AANA’s Code for Advertising and Marketing Communications to Children; the Australian Subscription Television and Radio Association (ASTRA) Code of Practice; and the Commercial Radio Code of Practice. The Children’s Television Standards (CTS) and the Commercial Television Industry Code of Practice (CTICP) include some restrictions regarding placement. For example, the CTS prohibits the broadcast of alcohol advertising during a ‘P’ program or period (suitable for pre-schoolers) and ‘C’ program or period (suitable for children ≤14 years of age) on free-to-air television. Broadcasters are required to show P and C programs for an average of one hour per day [6]; however, in practice this typically occurs outside of children’s peak viewing times [7].

### Issues Paper

In December 2012, the Australian National Preventive Health Agency (ANPHA) published an *Issues Paper* which reviewed current alcohol marketing regulations, focusing on children and young people’s exposure, and the effectiveness of these regulations in addressing community concerns about harmful alcohol consumption [8]. In particular, the ANPHA report examined: 1) the level of exposure to alcohol advertising among children and young people, for example, exposure arising from an exemption allowing alcohol advertisements to appear during live sport television broadcasts at times when alcohol advertising would ordinarily be banned; 2) the limited scope of current regulations, including new media marketing, the focus on content rather than placement, and the failure to regulate sponsorship of sporting and cultural events; 3) the voluntary nature of the current regulatory system; and, therefore, 4) its inability to penalise advertisers for breaches of the ABAC. Stakeholders were given the opportunity until March 2013 to present their views of current alcohol marketing regulations in submissions to ANPHA, the content of which is the subject of the current paper.

### Corporate Political Activity

There is a growing body of literature identifying vested interests as barriers to the implementation of effective public health policies [9–11]. Corporate political activity by the alcohol industry is a common strategy to influence policy in ways favourable to corporations [12, 13]. In Australia the alcohol industry is heavily involved in planning of public health policy; for instance, industry representatives were invited by the Inter-governmental Committee on Drugs to the development of a new National Drug Strategy [14]. However, research suggests that such partnerships advance the interests of the industry rather than public health [15, 16] because the industry merely argues the need for more research and promotes policies that fail to reduce alcohol sales, such as education and interventions aimed at only the riskiest drinkers [11, 17]. Illustrating the possible influence of alcohol industry is the comment reiterating industry claims by Fiona Nash, Assistant Minister for Health, in response to the release of the final ANPHA report on alcohol advertising: *“I do have concerns around the advertising of alcohol during sporting events*, *which is watched by many children…However the issue around it is genuinely complex and more research and work is required…”* [18].

Systematic analysis of alcohol industry framing of claims against increased marketing regulation has not been undertaken to date. Analysis of framing builds on notions of ‘agenda setting’ and ‘stakeholder analysis’. These approaches describe policy processes, but often neglect analysis of power and interests, and the strategies used to gain influence over policy, which are our focus. Framing analysis has a long history and has been used in different disciplines, for example, in cultural studies and communication [19, 20]; in sociology [21]; and in applied policy areas, such as environmental studies [22]. Its use in policy analysis of controversial policy issues draws on the work of Donald Schön and Martin Rein [23, 24], which has been applied to a diverse range of policies; and drawn on by others for interpretive policy analysis [25]. Framing the public health debate to align with commercial interests is one important industry strategy to influence policy makers and politicians [16]. This debate reflects the tension between personal freedom and collective responsibility [26] and represents two opposing ethical frames of (1) industry actors, asserting individual responsibility and limited government interference; and (2) public health actors, asserting the need for control of hazards, prevention of harm, and burden sharing [27, 28]. Policy makers are often unaware of the framing [29], so there is value in providing guidance to enable critique of alcohol industry framing of claims against the implementation of effective policies.

A recent study by Savell et al. [30] identified tactics and arguments used by the tobacco industry to influence policy on marketing regulation. Their work builds on research that applied corporate political analysis to wide-ranging policy applications [13]. Savell et al.’s review developed two frameworks to aid understanding of tobacco industry arguments and strategies. Given the parallels between tobacco and alcohol industry tactics to delay development of public health policy [31], we use the frameworks developed by Savell et al. to analyse the claims of the Australian alcohol industry in their submissions to the 2014 ANPHA issues report [30].

## Methods

### Procedure

We started with the assumption to usis the reader meant to make of this? er, cite evidence to support the claim.should be made.n of participants are likely to that corporations’ framing of alcohol problems, scientific evidence, and government policies, is part of a strategy to influence policies in ways likely to protect or generate profit.

We obtained all 34 submissions to the Australian National Preventative Health Taskforce Issues report [32]. We categorised the submissions in five stakeholder groups: 1) *alcohol industry*, including nine submissions from alcohol industry associations, major alcohol companies and retailers; 2) *media and marketing industry* (n = 9); 3) *public health*, including eight submissions from non-government organizations and academic research groups; 4) *governments* (n = 3); and, 5) *others* (n = 5), including three anonymous submissions (see Table 1).

**Table 1:** Submissions by stakeholder groups.

Stakeholder Group	N	Contributors (Referred to in this paper)
Alcohol industry (included in analysis)	9	• Winemakers' Federation of Australia (WFA) • Lion (Lion) • Diageo Australia (Diageo) • Brewers Association of Australia and New Zealand (BAANZ) • Australian Hotels Association (AHA) • Woolworths (Woolworths) • Australian Liquor Stores Association (ALSA) • Distilled Spirits Industry Council of Australia (DSICA) • Alcohol Beverages Advertising (and Packaging) Code Scheme (ABAC) ***(not included)***
Media and marketing industry	9	• Advertising Standards Bureau (ASB) • Commercial Radio Australia • The Publishers' Advertising Advisory Bureau (PAAB) • Australian Association of National Advertisers (AANA) • Interactive Advertising Bureau Australia (IAB) • Free TV Australia • Outdoor Media Association (OMA) • The Australian Subscription Television and Radio Association (ASTRA) • Association for Data-driven Marketing and Advertising
Non-government organizations/public health academics	8	• Victorian Alcohol and Drug Association (VAADA) • Cancer Council NSW • Ms Sarah Yeates, University of Queensland • Dr Nicholas Carah, University of Queensland / Dr Sven Brodmerkel, Bond University • Alcohol and other Drugs Council of Australia (ADCA)/ Public Health Association of Australia (PHAA) • National Alliance for Action on Alcohol (NAAA) • Foundation for Alcohol Research and Education (FARE) • McCusker Centre for Action on Alcohol and Youth (MCAAY)
Public servants	3	• Murrumbidgee Local Health District • Western Australian Police • Dr Adrian Reynolds, Department of Health, Tasmanian Government
Other (including 3 confidential submissions)	5	• Mr Sarosh Mehta • Foundation for Advertising Research (FAR)
Total	34	

Inclusion criteria for the analysis of submissions were: (1) authored by (or by a representative of) an alcohol industry association, an alcohol producer (or association), an alcohol retailer (or association), or an alcohol outlet (or association); and the content had to discuss ANPHA’s *Issues Paper*. Of the nine alcohol industry submissions, one simply provided information about the ABAC, its background, operations, services, management and coverage, and statistics about complaints and did not discuss the *Issues Paper*. Therefore, eight submissions by alcohol industry peak bodies, which broadly represent Australian manufacturers and retailers, were included.

### Savell et al.’s Corporate Political Activity Frameworks

Savell et al. developed classifications for the tobacco industry’s corporate political activity and split them into two frameworks. The first describes the attempts corporations made to influence marketing regulation, splitting them into *strategies* (such as *information*) and subcategories labelled *tactics* (such as contesting evidence; see S1 Table). The second is presented in terms of *frames* (such as *regulatory redundancy*) which included illustrative individual *arguments* (e.g., ‘the existing regulation is adequate’; see S2 Table) [30]. While Savell et al. use the term ‘arguments’ to refer to assertions or claims made by the tobacco industry in support of its position in favour of or against particular policies, we suggest a more suitable label. The word ‘argument’ is generally understood to refer to a connected series of propositions intended to establish a conclusion [33]. The validity of the conclusion depends on the veracity of the propositions and the soundness of the logic linking them. In our view, by using the term ‘argument’, Savell et al. elevate what are, almost without exception merely claims or assertions, to a status they do not deserve. Accordingly, we use the terms ‘claim’ and ‘assertion’ interchangeably in our analysis reflecting the class ‘argument’ in Savell et al.’s system.

Thematic analysis of these submissions was undertaken using deductive coding [34], according to Savell et al.’s *frames* and *arguments* (see S2 Table). As the current study concerns a different industry than was examined by Savell et al., emergent coding (an inductive approach) was also used to adapt and develop new categories specific to the alcohol industry [35]. Thus, an integrated approach involving inductive and deductive methods was used to develop a categorization of *frames and claims* [36]. Final categories were decided on once all the submissions were coded independently by two researchers (FM and KC). Inter-coder reliability was strengthened by evolving decision rules for coding where categories were cross-checked and amended as appropriate and the addition of new tactics where necessary. After independently coding the data according to the (deductive) Savell et al. coding framework, the two coders had in-depth discussion to establish consensus on appropriate frames and claims.

## Results

### Frames and Claims

We identified the same four frames as those in Savell et al.’s analysis of tobacco industry behaviour: 1) *Regulatory Redundancy*; 2) *Insufficient Evidence*; 3) *Negative Unintended Consequences*; and 4) *Legal*. In addition, we identified 5) *Corporate Social Responsibility* as a frame. Within these five frames we identified several other types of claims in addition to those identified by Savell et al., especially under *Regulatory Redundancy*; and adapted other existing claims (see Table 2 for summary of frames and claims).

**Table 2:** Claims used by the alcohol industry attempting to influence marketing regulation using Savell et al.’s classification framework.

**Frame**		**Sub-frames (where applicable)**				**Claims in submissions (number of submissions presenting claim, out of 8)**		**Example quote from the submissions**		**Assigned quote number‡**	
Regulatory Redundancy (8)						Industry only markets to those of legal age/is actively opposed to minors using product (4)		*“Alcohol is a legal product and individual producers are well within their rights to use advertising for commercial gain provided the activities do not promote misuse and meet ABAC requirements*.*”* (WFA)		*1*	
						Current self-regulation is satisfactory^#^ (8)		*“The independent pre-vetting service and the adjudication process for handling complaints are particularly effective in stopping irresponsible marketing*.*”* (Diageo)		*2a*	
								*“The ABAC Scheme is flexible to changing marketing conditions and techniques*, *and can quickly respond to new marketing developments*.*”* (DSICA)		2b	
						Industry adheres to own self-regulatory codes (5)		*“ABAC has the support and backing of the alcohol and advertising industries*, *which reduces the level of ‘gaming’ that can take place with regulation that relies on ‘black letter law’ and strict definitions*.*”* (DSICA)		*3*	
						Codes are supported by the government* (4)		*“It has continuous and substantial input from the Australian Government*.*”* (DSICA)		*4*	
						Most consumers drink responsibly* (6)		*“The vast majority of Australians enjoy alcohol responsibly*.*”* (ALSA)		*5*	
						Drinking is part of a healthy lifestyle* (4)		*“…moderate consumption of alcohol*, *which is a normal*, *enjoyable part of life for many adults*.*”* (Lion)		*6a*	
								*“…when consumed in moderation*, *[alcohol] can be part of a healthy*, *balanced lifestyle*.*”* (WFA)		*6b*	
						Suggesting alternative policy strategies that address harmful consumption of minority that misuses alcohol * (6)		*“Alcohol policies that seek to reduce total alcohol consumption in Australia will not reduce misuse*, *but rather simply punish the majority of consumers who are already drinking responsibly in moderation*.*”* (BAANZ)		*7a*	
								*”…the most effective way to reduce harmful consumption of alcohol is a focus on targeted interventions as opposed to any further population wide restrictions*.* *.* *.*”* (BAANZ)		*7b*	
						Disputing community concern/ codes are in line with community expectations* (8)		*“…the complaints process and code accurately delivers against broader community expectations”*. (Diageo)		*8a*	
								*“The small percentage of alcohol advertisements complained about each year…reinforces the industry view that there is no widely held community concern about alcohol advertising…”* (WFA)		*8b*	
								*“The AHA cautions against overstating community concern…”* (AHA)		*8c*	
						Alcohol industry encourages responsible consumption* (4)		*“Responsible drinking is at the heart of our business interests*.*”* (Diageo)		*9a*	
								*“…committed to working with ANPHA and others to better understand and develop strategies to address such problems…”* (Lion)		*9b*	
Insufficient Evidence (8)				There’s insufficient evidence that the proposed policy will work / marketing doesn’t increase overall consumption levels (marketing is used to convince individuals switch brands and to sustain or increase company’s market share), so regulation will have no effect^#^ (8)		More research is needed, insufficient evidence for causal link between marketing and increased consumption levels (5)		*“Important areas of contention*, *such as the link between advertising and misuse*, *require further analysis and for a clear consensus to emerge in the relevant research*.*”* (WFA)		*10*	
						Marketing only affects market share (5)		*“Diageo markets its brands […] to gain market share by encouraging consumers to switch from other brands to one of ours*. *Our marketing is not designed to increase overall consumption of alcohol*.*”* (Diageo)		*11*	
						Reporting on declining trends of alcohol consumption (5)		*“…in fact alcohol misuse has declined in Australia over the last few decades*.*”* (Lion)		*12a*	
								*“The case for further restrictions on alcohol advertising is further weakened when looking more broadly at per capita consumption of alcohol as this has been essentially static for the past 20 years*. *If advertising increases alcohol consumption then it does not appear to have had any impact in Australia*.*”* (DSICA)		*12b*	
						Biased public health advocates* (6)		*“The present structure for administering the ABAC Scheme has not attracted criticisms other than from individuals or organizations that have taken a very public anti-alcohol or anti-industry position with questionable motives*.*”* (ALSA)		*13*	
Negative Unintended Consequences (6)		Economic (6)		Manufacturer (4)		Regulation will cause problems maintaining or increasing market share for existing brands^#^ (3)		*“It would also have the potential to introduce significant market distortion to the competition between responsible producers trying to win market share*.*”* (WFA)		*14*	
						Regulation will cause difficulties for new market entrants^#^ (1)		*“New market entrants will find it much more difficult to establish a presence if advertising is restricted*, *creating significant competition implications*.*”* (Lion)		*15*	
				Associated Industries (4)		Regulation will result in financial or job losses (among retailers or associated industries, e.g. agriculture, hospitality, tourism, manufacturing and logistics) (4)		*“Placing further*, *more onerous restrictions on advertisers will have a serious commercial impact on a wide range of industries…”* (Lion)		*16a*	
								*“…due to [the alcohol industry’s] important role in the agricultural*, *brewing*, *tourism and hospitality sectors…”* (BAANZ)		*16b*	
				Public Revenue (2)		Loss of direct contribution to the Australian economy by alcohol industry^#^ (2)		*“ACIL Tasman has estimated that the direct economic contribution of the Australian brewing industry to the Australian economy was approximately $4*.*3 billion in the 2010–11 financial year*.*”* (BAANZ)		*17*	
				Consumers* (3)		Impacts on consumer choice* (3)		*“Without the ability to be informed of their choices*, *consumers suffer a loss of welfare as they are not aware of new products…”* (DSICA)		*18*	
		Public Health (1)				Regulation might impact negatively on health outcomes in moderate drinkers^#^ (1)		*“…alcohol policy should not impact moderate drinkers in its efforts to address problem drinkers*, *as this will result in perverse health outcomes*.*”* (Lion)		*19*	
Legal (5)						Regulatory Impact Statement (RIS) needs to be developed before proposing new regulation* (3)		*“Further consideration of the potential increased regulatory burden on the industry from the proposals canvassed in the Issues Paper also demands a Regulatory Impact Statement process*.*”* (WFA)		*20*	
						Body does not have the power to regulate/it’s beyond their jurisdiction (3)		*“The AHA is also surprised to see in the Issues Paper a significant broadening of scope beyond that directed to ANPHA in the Australian Government Response to the Preventative Health Taskforce Report…”* (AHA)		*21*	
Corporate Social Responsibility* (6)						Supporting efforts and programs to reduce harmful consumption* (5)		*“Recent examples of our social responsibility initiatives include…a social marketing campaign*, *using the strapline ‘Don’t see a good night wasted’*, *aimed at 18–25 year olds socializing in and around licensed venues in Sydney*.*”* (Diageo)		*22*	
						We are members of DrinkWise *(4)		*“Lion is also a founding member of DrinkWise Australia…”* (Lion)		*23a*	
								*“Woolworths fully supports the efforts and activities of DrinkWise that aim to affect generational change in the way all Australians consume alcohol*.*”* (Woolworths)		*23b*	
**Frame**		**Sub-frames (where applicable)**				**Claims in submissions (number of submissions presenting claim, out of 8)**		**Example quote from the submissions**		**Assigned quote number‡**	
Regulatory Redundancy (8)						Industry only markets to those of legal age/is actively opposed to minors using product (4)		*“Alcohol is a legal product and individual producers are well within their rights to use advertising for commercial gain provided the activities do not promote misuse and meet ABAC requirements*.*”* (WFA)		*1*	
						Current self-regulation is satisfactory^#^ (8)		*“The independent pre-vetting service and the adjudication process for handling complaints are particularly effective in stopping irresponsible marketing*.*”* (Diageo)		*2a*	
								*“The ABAC Scheme is flexible to changing marketing conditions and techniques*, *and can quickly respond to new marketing developments*.*”* (DSICA)		2b	
						Industry adheres to own self-regulatory codes (5)		*“ABAC has the support and backing of the alcohol and advertising industries*, *which reduces the level of ‘gaming’ that can take place with regulation that relies on ‘black letter law’ and strict definitions*.*”* (DSICA)		*3*	
						Codes are supported by the government* (4)		*“It has continuous and substantial input from the Australian Government*.*”* (DSICA)		*4*	
						Most consumers drink responsibly* (6)		*“The vast majority of Australians enjoy alcohol responsibly*.*”* (ALSA)		*5*	
						Drinking is part of a healthy lifestyle* (4)		*“…moderate consumption of alcohol*, *which is a normal*, *enjoyable part of life for many adults*.*”* (Lion)		*6a*	
								*“…when consumed in moderation*, *[alcohol] can be part of a healthy*, *balanced lifestyle*.*”* (WFA)		*6b*	
						Suggesting alternative policy strategies that address harmful consumption of minority that misuses alcohol * (6)		*“Alcohol policies that seek to reduce total alcohol consumption in Australia will not reduce misuse*, *but rather simply punish the majority of consumers who are already drinking responsibly in moderation*.*”* (BAANZ)		*7a*	
								*”…the most effective way to reduce harmful consumption of alcohol is a focus on targeted interventions as opposed to any further population wide restrictions*.* *.* *.*”* (BAANZ)		*7b*	
						Disputing community concern/ codes are in line with community expectations* (8)		*“…the complaints process and code accurately delivers against broader community expectations”*. *(Diageo)*		*8a*	
								*“The small percentage of alcohol advertisements complained about each year…reinforces the industry view that there is no widely held community concern about alcohol advertising…”* (WFA)		*8b*	
								*“The AHA cautions against overstating community concern…”* (AHA)		*8c*	
						Alcohol industry encourages responsible consumption* (4)		*“Responsible drinking is at the heart of our business interests*.*”* (Diageo)		*9a*	
								*“…committed to working with ANPHA and others to better understand and develop strategies to address such problems…”* (Lion)		*9b*	
Insufficient Evidence (8)				There’s insufficient evidence that the proposed policy will work / marketing doesn’t increase overall consumption levels (marketing is used to convince individuals switch brands and to sustain or increase company’s market share), so regulation will have no effect^#^ (8)		More research is needed, insufficient evidence for causal link between marketing and increased consumption levels (5)		*“Important areas of contention*, *such as the link between advertising and misuse*, *require further analysis and for a clear consensus to emerge in the relevant research*.*”* (WFA)		*10*	
						Marketing only affects market share (5)		*“Diageo markets its brands […] to gain market share by encouraging consumers to switch from other brands to one of ours*. *Our marketing is not designed to increase overall consumption of alcohol*.*”* (Diageo)		*11*	
						Reporting on declining trends of alcohol consumption (5)		*“…in fact alcohol misuse has declined in Australia over the last few decades*.*”* (Lion)		*12a*	
								*“The case for further restrictions on alcohol advertising is further weakened when looking more broadly at per capita consumption of alcohol as this has been essentially static for the past 20 years*. *If advertising increases alcohol consumption then it does not appear to have had any impact in Australia*.*”* (DSICA)		*12b*	
						Biased public health advocates* (6)		*“The present structure for administering the ABAC Scheme has not attracted criticisms other than from individuals or organizations that have taken a very public anti-alcohol or anti-industry position with questionable motives*.*”* (ALSA)		*13*	
Negative Unintended Consequences (6)		Economic (6)		Manufacturer (4)		Regulation will cause problems maintaining or increasing market share for existing brands^#^ (3)		*“It would also have the potential to introduce significant market distortion to the competition between responsible producers trying to win market share*.*”* (WFA)		*14*	
						Regulation will cause difficulties for new market entrants^#^ (1)		*“New market entrants will find it much more difficult to establish a presence if advertising is restricted*, *creating significant competition implications*.*”* (Lion)		*15*	
				Associated Industries (4)		Regulation will result in financial or job losses (among retailers or associated industries, e.g. agriculture, hospitality, tourism, manufacturing and logistics) (4)		*“Placing further*, *more onerous restrictions on advertisers will have a serious commercial impact on a wide range of industries…”* (Lion)		*16a*	
								*“…due to [the alcohol industry’s] important role in the agricultural*, *brewing*, *tourism and hospitality sectors…”* (BAANZ)		*16b*	
				Public Revenue (2)		Loss of direct contribution to the Australian economy by alcohol industry^#^ (2)		*“ACIL Tasman has estimated that the direct economic contribution of the Australian brewing industry to the Australian economy was approximately $4*.*3 billion in the 2010–11 financial year*.*”* (BAANZ)		*17*	
				Consumers* (3)		Impacts on consumer choice* (3)		*“Without the ability to be informed of their choices*, *consumers suffer a loss of welfare as they are not aware of new products…”* (DSICA)		*18*	
		Public Health (1)				Regulation might impact negatively on health outcomes in moderate drinkers^#^ (1)		*“…alcohol policy should not impact moderate drinkers in its efforts to address problem drinkers*, *as this will result in perverse health outcomes*.*”* (Lion)		*19*	
Legal (5)						Regulatory Impact Statement (RIS) needs to be developed before proposing new regulation* (3)		*“Further consideration of the potential increased regulatory burden on the industry from the proposals canvassed in the Issues Paper also demands a Regulatory Impact Statement process*.*”* (WFA)		*20*	
						Body does not have the power to regulate/it’s beyond their jurisdiction (3)		*“The AHA is also surprised to see in the Issues Paper a significant broadening of scope beyond that directed to ANPHA in the Australian Government Response to the Preventative Health Taskforce Report…”* (AHA)		*21*	
Corporate Social Responsibility* (6)						Supporting efforts and programs to reduce harmful consumption* (5)		*“Recent examples of our social responsibility initiatives include…a social marketing campaign*, *using the strapline ‘Don’t see a good night wasted’*, *aimed at 18–25 year olds socializing in and around licensed venues in Sydney*.*”* (Diageo)		*22*	
						We are members of DrinkWise *(4)		*“Lion is also a founding member of DrinkWise Australia…”* (Lion)		*23a*	
								*“Woolworths fully supports the efforts and activities of DrinkWise that aim to affect generational change in the way all Australians consume alcohol*.*”* (Woolworths)		*23b*	

* Frame or claim developed by Martino et al. 2014

^#^ Frame or claim taken from Savell et al. and adapted for the alcohol industry by Martino et al. 2014

‡Researcher assigned quote number; referred to in the results section text

The following claims, taken from Savell et al.’s corporate political activity framework, were not used by the Australian Alcohol Industry: The health impacts of consumption remain unproven; The cost of compliance for manufacturers will be high/the time required for implementation has been underestimated; Regulation will cause an increase in illicit trade; Regulation could have other negative unintended consequences; Infringes legal rights of company (trademarks, intellectual property etc.); Regulation is more extensive than necessary/regulation is disproportionate; Regulation will cause an increase in compensation claims.

#### Regulatory redundancy

Submissions asserted that because it is a legal product alcohol is legitimately advertised to adults (e.g., #1, refers to exemplar quotes provided in Table 2). They also claimed that the current system is satisfactory, that self-regulation is flexible and responsive, and that social marketing is sufficiently regulated by this mechanism (eg #2a, b); that the public complaint system is accessible; and that the adjudication panel and pre-vetting experts are independent. Some businesses claimed to have gone further and developed their own codes and guidelines that operate alongside the existing marketing codes (eg #3). Another type of industry claim was that they have ongoing ‘partnerships’ with Australian governments, via their representation on the ABAC management committee (eg #4). Some called the system ‘quasi-‘ or ‘co-regulation’ instead of self-regulation. Relatedly, different types of claims were that ‘the vast majority of people drink responsibly’ (eg #5), and that ‘drinking alcohol can be part of a healthy lifestyle’ (eg #6a, b).

Other submissions included assertions disputing the increasing community concern about the link between alcohol advertising and risky drinking (eg #8a, b, 9a, b). Finally, submissions claimed that one of the industry’s goals is to promote ‘responsible consumption of alcohol’ (eg #9a, b).

#### Insufficient evidence

It was claimed within industry submissions that there was insufficient evidence to link marketing of alcohol products to increased alcohol consumption, and therefore, that marketing regulation would have no effect (eg #10). Some specifically stated that more research would be needed to prove this link. Submissions cited Australian government research purporting to show a decline in alcohol consumption in minors and pregnant women [37] and claiming *“…there is no evidence to suggest that alcohol problems are on the rise which could justify further regulatory constraints on the alcohol industry”* (Brewers Association of Australia and New Zealand (BAANZ)).

*Biased public health advocates* was a newly identified type of claim within the *Insufficient Evidence* frame. Submissions asserted that the Expert Committee on Alcohol, with whom ANPHA consulted to develop this report, was biased and anti-alcohol and that the research referenced in the report was not scientifically valid (eg #13). For example: *“Lion believes that ANPHA should be careful to distinguish between research that is the best available*, *expert*, *peer-reviewed research and surveys that are produced by anti-alcohol activists…”* (Lion).

#### Negative unintended consequences

A set of claims was also framed around the notion that increased regulation has *negative unintended consequences*. The key themes were: 1) manufacturers, who would, as consequence of regulation, have trouble maintaining or increasing market share (eg #14), or have difficulties introducing new brands (eg #15); 2) employment in associated industries (eg #16a, b); 3) loss of public revenue from alcohol tax and the alcohol industry’s direct contribution to the Australian economy (eg #17); and 4) loss of consumer sovereignty (eg #18). In contrast to the tobacco industry, no submissions mentioned ‘Illicit Trade’ and only one warned of *“…perverse health outcomes”* of increased marketing regulation, without explaining what these were (eg #19).

#### Legal

Two claims were identified within the *Legal* frame. Some submissions asserted the need for a Regulatory Impact Statement before proposing new regulation, for example *“…any proposals to further regulate alcohol advertising needs to clearly demonstrate that the social and economic cost it potentially introduces are outweighed by the benefits in an environment where rates of “at risk” consumption and harm are either stable or in decline*.*”* (#20, Winemaker’s Federation of Australia). A number of submissions questioned why alcohol marketing regulation was reviewed in the first place, as it was, according to them, not ANPHA’s task to do this (eg #21). Unlike Savell et al.’s findings regarding the tobacco industry, the alcohol industry did not refer to international trade agreements or intellectual property.

#### Corporate Social Responsibility

Some submitters claimed they were ‘socially responsible companies’ by presenting involvement in efforts and programs to reduce harmful consumption. For instance, *“Recent examples of our social responsibility initiatives include…a social marketing campaign*, *using the strapline ‘Don’t see a good night wasted’*, *aimed at 18–25 year olds socializing in and around licensed venues in Sydney*.*”* (#22; Diageo). Some emphasised their membership of DrinkWise (eg #23a; an industry funded ‘social aspects/public relations’ organization (SAPRO)), as evidence of their commitment to *Corporate Social Responsibility* [11]. For example, Lion stated that it *“…is committed to…funding culture change initiatives*, *such as those developed by DrinkWise…”*.

#### Alternative Strategies

Submissions provided recommendations for alternative strategies that the government could use to address the small section of society that drinks heavily, instead of *“punishing the majority”* of responsible drinkers (BAANZ). Proposed alternative countermeasures focused on individual responsibility, for example, education, and more severe drink-driving penalties.

## Discussion

The Australian alcohol industry used the following five overarching frames to oppose increased alcohol marketing regulation: 1) *Regulatory Redundancy*; 2) *Insufficient Evidence*; 3) *Negative Unintended Consequences*; 4) *Legal;* and 5) *Corporate Social Responsibility*. Savell et al.’s tobacco industry corporate political activity framework for frames and arguments was, for the most part, applicable to the analysis of the Australian alcohol industry policy documents with one additional frame needed to characterise the submissions, namely: *Corporate Social Responsibility*. The predominant alcohol industry claims were that increased marketing regulation was unnecessary in Australia and that there is insufficient evidence to support the proposal to regulate the promotion of alcohol. In contrast, the tobacco industry focused more on supposed detrimental economic and legal effects of regulation [30]. These findings reflect the different stages of government regulation applied to these two industries (i.e., their different positions on the regulatory pyramid), where tobacco is regulated more strictly by legislation than alcohol, where industry codes prevail [38]. Tobacco marketing in many countries is heavily regulated and evidence of the effectiveness of these comprehensive policies is plentiful [39, 40], such that the tobacco industry focuses on the negative economic effects of such regulation. Few governments, on the other hand, are actively considering stronger alcohol marketing regulation and the alcohol industry argues that current self-regulation is working well [41].

### Regulatory Redundancy

We identified nine different claims within the *Regulatory Redundancy* frame, whereas Savell et al. found only three used by the tobacco industry. The alcohol industry claims its marketing targets only adults, however, research shows that young people are also exposed to this marketing and are negatively affected by it [1, 2]. If the industry genuinely wishes to target only adults, the self-regulatory codes should include restrictions on sport sponsorship, outdoor media and product placement in films and music videos.

While the ABAC scheme has a high voluntary participation rate [42], the support may reflect the low standards of the code, the low likelihood of a finding against advertisers, and the lack of penalties in the event of complaints being upheld [15]. The submissions cited the low number of complaints submitted to ABAC as evidence of no community concern about the marketing of alcohol to children. However, an alternative complaint panel, set up by the McCusker Centre for Action on Alcohol and Youth and Cancer Council WA, the Alcohol Advertising Review Board, received more than double the number of complaints in their first year (2012), 68% of which were upheld, compared with only 7% of those considered by the ABAC [42].

The alcohol industry repeats the mantras that ‘most people drink responsibly’ and that alcohol consumption can be ‘part of a healthy lifestyle’ [43], claiming then that the majority of the population should therefore not be ‘punished for the sins of the few’ through policies that reduce the promotion of alcohol [44]. In line with this, the alcohol industry promotes targeted regulation for the ‘minority of problematic drinkers’ [45]. Research shows that alcohol marketing has a deleterious effect on vulnerable groups, such as ethnic minorities and problem drinkers [46], and these groups are specifically targeted through segment marketing [47]. Further, alcohol marketing has implications beyond these minority groups for adults in general [48, 49], underlining the need for broad restrictions.

### Insufficient Evidence

Like the tobacco industry, the alcohol industry proposed that there is insufficient evidence to show that marketing influences consumption, asserting that it merely affects brand loyalty. However, a recent analysis of alcohol industry documents shows that the major companies plan to create new drinking occasions and opportunities, that is, to increase overall consumption [50]. Recently, Ross et al. [51] found that, after controlling for variables known to influence drinking rates, such as parental drinking and overall market share, minors drink the brands they see advertised most. The industry argued in the submissions that there is insufficient evidence on the effectiveness of increased regulation on consumption levels, however, a recent extensive cross-national study showed that higher levels of regulation in Europe were associated with lower consumption in adults [52].

### Negative Unintended Consequences

Savell et al. reported that the tobacco industry framed most of its claims around negative unintended consequences of increased marketing regulation, whereas the alcohol industry submissions in this study presented few such claims. Some asserted that alcohol production provides substantial economic benefit and employment within Australia, and that increased marketing regulation would adversely affect the economy. While effective regulation would undoubtedly reduce alcohol production and marketing, such impacts should be considered in light of changes related to global market conditions and in light of the cost of alcohol harm. For example, the recently announced merger of AB InBev and SABMiller, will result in ‘savings’ to the company of $1.4b, most of which is being achieved by exporting jobs to countries where wages are lower [53]. On the other hand, the reductions in potential sales are comparatively small when compared to the huge direct societal cost of alcohol consumption to the Australian community, estimated to be $14.352b in 2010 alone [54].

In contrast to tobacco submissions, alcohol industry submissions did not mention illicit trade, and only one mentioned negative public health consequences (Lion). While the alcohol industry is still defending self-regulation, the tobacco industry seems to accept that it has lost this battle and therefore focuses on economic consequences of increased regulation.

### Legal

Savell et al. [30] identified four different claims used by the tobacco industry under the *Legal* frame; namely, that restrictions are infringements of legal rights (for example trademarks), that they constitute disproportionate regulation, that the body in question does not have the power to regulate, and that there would be an increasing number of compensation claims. We found only two alcohol industry assertions within this frame, namely, the need for *Regulation Impact Statements* and that ANPHA does not have the power to regulate. The Australian Government requires that a *Regulation Impact Statements* is prepared for significant regulatory proposals [55], however, ANPHA’s role was to provide policy advice to the Department of Health, not to put a proposal to Cabinet, and it was therefore not appropriate for ANPHA to provide such a statement. Contesting the authority of key organizations or groups involved in policy development, such as ANPHA, is common practice of ‘dangerous consumption’ industries [30]. In this case, however, Australia’s Intergovernmental Committee on Drugs underpins ANPHA’s role legitimacy: *“…it was decided that ANPHA’s approach to alcohol advertising should be broadened to review the effectiveness of the alcohol industry’s voluntary code on advertising and its effectiveness in addressing community concerns”* [56]. The three other claims within Savell et al.’s frame were not identified in the current study, probably because the threat of legislation is lower than it is for the tobacco industry.

### Corporate Social Responsibility

*Corporate Social Responsibility* was invoked in submissions to encourage policy advisors to resist recommending regulation of alcohol marketing. A recent UK study confirms that corporate social responsibility initiatives are a vehicle for the alcohol industry to influence government policy [17]. Another study of British American Tobacco’s internal documents showed that corporate social responsibility initiatives are a key corporate political activity, because they facilitate access to policy makers [57]. For the alcohol industry, stating in a submission that the company is a member of DrinkWise has also been identified as a tactic to establish submitters’ credentials as socially responsible corporations [11].

### Accusations of Bias

Finally, *Biased Public Health Advocates* was a newly identified type of claim within the frame of *Insufficient Evidence*. While actors with vested interests have often engaged in disputes with advocates for evidence-based reform, there has rarely been a focus on public health advocates in official documents. Attacking the credibility of public health advocates in submissions to government appears to be increasing [58]. Rosenstock and colleagues proposed that government agencies, academic centres, and researchers affiliated with them are subject to efforts to politicise or silence independent scientific researchers. Such efforts may employ sophisticated strategies that put evidence-based policy making at risk, especially because most researchers are not trained or prepared for such attacks, and most are unable to access policy-makers to the extent that corporate lobbyists can. This appears to be an extension of the tactics employed by as the likes of the tobacco industry which support movements such as ‘Junk science’ to undermine public and political confidence in science [59, 60]. This tactic deserves further investigation as previous literature demonstrates that such strategies are usually part of sophisticated, well-resourced, and outcome-focused campaigns [61].

## Limitations

We sought to minimise bias in the subjectivity of thematic coding by having two researchers (FM and KC) independently code all documents, and after discussion, reaching agreement on all thematic classification. They discussed and decided on adaptations of existing classification frameworks, and created new frames and claims to characterise the alcohol industry submissions. A second limitation is the single country focus. Savell et al. [30] showed that the tobacco industries worldwide use coherent strategies and claims to influence marketing regulation. It remains unknown as to whether alcohol industry bodies in other countries adopt similar strategies [62].

## Conclusions

This study examined Australian alcohol industry claims regarding marketing regulation, finding strong similarities with the frames and claims used by the tobacco industry [30]. Alcohol industry actors used multiple strategies to push their claims that increased marketing regulation in Australia is unnecessary, including claims that: there is ‘insufficient evidence for the effectiveness of increased regulation’; ‘there is insufficient evidence that alcohol marketing contributes to drinking’; ‘current regulation is satisfactory’; ‘there is no community concern’; and that ‘the alcohol industry markets its products in a way that minimise harmful consumption’. These assertions, at least regarding health, stand in contrast to the scientific literature regarding alcohol-related harm and continuing high levels of alcohol consumption in the community. The science reveals the poverty of industry claims that industry actors put to public servants whose job it is to evaluate submissions. Recent tobacco research [63] suggests that the tobacco industry seeks to ‘sow reasonable doubt’ about the science [64] among policy makers in order to resist or delay regulation. Continuing to engage with industry as stakeholders in public health policies increases their opportunities to present such claims [63].

## Supporting Information

S1 Table Tactics used by the Tobacco Industry when attempting to influence marketing regulation (DOCX)

S2 Table Arguments used by the Tobacco Industry when attempting to influence marketing regulation (DOCX)
