# Correction: Analysis of Alcohol Industry Submissions against Marketing Regulation

**DOI:** 10.1371/journal.pone.0175661

**Published:** 2017-04-06

**Authors:** Florentine Petronella Martino, Peter Graeme Miller, Kerri Coomber, Linda Hancock, Kypros Kypri

There is an error in the first sentence of the Methods section. The correct sentence is: We started with the assumption that corporations’ framing of alcohol problems, scientific evidence, and government policies, is part of a strategy to influence policies in ways likely to protect or generate profit.
