# Supplementary material for: Analysis of Alcohol Industry Submissions against Marketing Regulation
Source: PLoS One. 2017 Jan 24;12(1):e0170366. doi: 10.1371/journal.pone.0170366 (PMC5261775; doi:10.1371/journal.pone.0170366)
Supplement: S1 Table — (DOCX) [file pone.0170366.s001.docx]

**Supporting Information**

**S1 Table. Tactics used by the Tobacco Industry when attempting to influence marketing regulation**

| **Strategy** | **Tactic** | |
| --- | --- | --- |
| Information | Direct lobbying (meetings and correspondence with legislators/policymakers) | |
|  | Indirect lobbying (using third parties, including front groups, to lobby on the industry’s behalf) | |
|  | Shaping the evidence base | Commissioning, writing (or ghost writing), or disseminating research/publications |
|  |  | Preparing position papers, technical reports or data on impacts  (including economic impact studies) |
|  | Establishing industry/policymaker collaboration (e.g. via working group, technical group, advisory group)/work alongside policymakers providing technical support/advice | |
| Constituency building | External constituency building | Form alliances with and mobilise other industry sectors/business/trade organisations |
|  |  | Media advocacy (press releases, publicity campaigns, public hearings, interviews) |
|  |  | Form alliances with or mobilize unions/civil society organizations/ consumers/employees/the public |
|  |  | Creation of front groups or astroturf organisations |
|  | Internal constituency building | Collaboration between companies/development of pan-industry group or industry trade association |
| Policy substitution | Develop/promote (new or existing) voluntary code/self-regulation | |
|  | Develop/promote alternative regulatory policy | |
|  | Develop/promote non-regulatory initiative (generally seen to be ineffective/less effective, e.g. education programmes) | |
| Legal | Pre-emption | |
|  | Using litigation/threat of legal action | |
| Constituency fragmentation  and destabilization | Preventing the emergence of, neutralising and/or discrediting potential opponents (individuals, organisations or coalitions) | |
| Financial Incentive | Providing current or offering future employment to those in influential role | |
|  | Gifts, entertainment or other direct financial inducement | |

**Source: Savell E, Gilmore AB, Fooks G (2014) How Does the Tobacco Industry Attempt to Influence Marketing Regulations? A Systematic Review. PLoS ONE 9(2): e87389. Table 2**
