# Supplementary material for: Analysis of Alcohol Industry Submissions against Marketing Regulation
Source: PLoS One. 2017 Jan 24;12(1):e0170366. doi: 10.1371/journal.pone.0170366 (PMC5261775; doi:10.1371/journal.pone.0170366)
Supplement: S2 Table — (DOCX) [file pone.0170366.s002.docx]

**S2 Table. Arguments used by the Tobacco Industry when attempting to influence marketing regulation.**

| **Frame** | **Sub-frames (where applicable)** | | **Argument** | **Argument/claims observed in Alcohol industry? (Yes/No)** |
| --- | --- | --- | --- | --- |
| Negative Unintended Consequences | Economic | Manufacturer | The cost of compliance for manufacturers will be high/the time required for implementation has been underestimated | No |
|  |  |  | Regulation will result in financial or job losses (among manufacturers) | Yes |
|  |  |  | The regulation is discriminatory/regulation will not affect all producers/customers equally | Yes |
|  |  | Public Revenue | Regulation will cause economic/financial problems (for city, state, country or economic area (e.g. European Union)) | Yes |
|  |  | Associated Industries | Regulation will result in financial or job losses (among retailers and other associated industries, e.g. printing, advertising, leisure) | Yes |
|  | Public Health |  | Regulation will have negative public health consequences | Yes |
|  | Illicit Trade |  | Regulation will cause an increase in illicit trade | No |
|  | Other |  | Regulation could have other negative unintended consequences (e.g. cause confusion amongst customers, set a precedent for other types of products/’slippery slope’) | No |
| Legal |  |  | Infringes legal rights of company (trademarks, intellectual property, constitutionally protected free speech (e.g. US First Amendment), international trade agreements) | No |
|  |  |  | Regulation is more extensive than necessary/regulation is disproportionate | No |
|  |  |  | Body doesn’t have the power to regulate/it’s beyond their jurisdiction | Yes |
|  |  |  | Regulation will cause an increase in compensation claims | No |
| Regulatory Redundancy |  |  | Industry adheres to own self-regulation codes/self-regulation is working well | Yes |
|  |  |  | Industry only markets to those of legal age/is actively opposed to minors using product | Yes |
|  |  |  | Existing regulation is satisfactory/existing regulation is satisfactory, but requires better enforcement | Yes |
| Insufficient Evidence |  |  | There’s insufficient evidence that the proposed policy will work / marketing doesn’t cause or change behavior (it’s only used for brand selection and capturing market share), so regulation will have no effect | Yes |
|  |  |  | The health impacts of consumption remain unproven | No |

**Source: Savell E, Gilmore AB, Fooks G (2014) How Does the Tobacco Industry Attempt to Influence Marketing Regulations? A Systematic Review. PLoS ONE 9(2): e87389. Table 3.**
